# Current guidelines on the management of gestational diabetes mellitus: a content analysis and appraisal

**DOI:** 10.1186/s12884-019-2343-2

**Published:** 2019-06-13

**Authors:** Mengxing Zhang, Yingfeng Zhou, Jie Zhong, Kairong Wang, Yan Ding, Li Li

**Affiliations:** 1 Fudan University Centre for Evidence-based Nursing: A Joanna Briggs Institute Centre of Excellence, School of Nursing, Fudan University, Shanghai, China; 2 Obstetrics and Gynecology Hospital, Fudan University, Shanghai, China

**Keywords:** Gestational diabetes mellitus, Content analysis method, Clinical practice guideline, Recommendation matrix

## Abstract

**Background:**

Despite many guidelines for the management of gestational diabetes available internationally, little work has been done to summarize and assess the content of existing guidelines. A paucity of analysis guidelines within in a unified system may be one explanatory factor. So this study aims to analyze and evaluate the contents of all available guidelines for the management of gestational diabetes.

**Method:**

Relevant clinical guidelines were collected through a search of relevant guideline websites and databases (PubMed, Web of Science, Embase, etc.). Fourteen guidelines were identified, and each guideline was assessed for quality using the Appraisal of Guidelines for Research & Evaluation (AGREE) II instrument. Two independent reviewers extracted guideline recommendations using a “recommendation matrix” through which basic guideline information and consistency between search strategy and selection of evidence, between selected evidence and interpretation, and between interpretation and resulting recommendations were analyzed.

**Results:**

Fourteen documents were analyzed, and a total of 361 original recommendations for gestational diabetes mellitus (GDM) management were assessed. In all guidelines included, the recommendations were developed in five domains, namely, diagnosis of GDM, prenatal care, intrapartum care, neonatal care and postpartum care. Different guidelines appeared to have significant discrepancy in consistency of guideline content, but overall, there was consistency between search strategy and selection of evidence, between selected evidence and interpretation, and between interpretation and resulting recommendations (scilicet 49.31, 57.20 and 58.17%, respectively).

**Conclusion:**

Although commonality in most recommendations existed, there were still some discrepancies between guidelines. Consistency of guidelines on the management of GDM in pregnancy is highly variable and needs to be improved.

**Electronic supplementary material:**

The online version of this article (10.1186/s12884-019-2343-2) contains supplementary material, which is available to authorized users.

## Background

Gestational diabetes mellitus (GDM) is a special form of diabetes in women of child-bearing age and is a common gestational endocrine disease [1]. Due to its increasing prevalence, GDM results in significant short- and long-term impairments in the individual’s health and their offspring’s health [2–6]. Consistent evidence from high-quality randomized controlled trials over the last few decades has determined that proper management is effective in ensuring pregnancy outcomes and long-term outcomes in GDM women [7, 8]. However, management of GDM in the real world of clinical practice seems to be unsatisfactory [9], so it is necessary to standardize the management of GDM.

Clinical practice guidelines (CPGs) are statements that include recommendations intended to assist providers and recipients of healthcare and other stakeholders to make informed decisions, and they are effective tools for disseminating medical knowledge [10]. With regard to the management of GDM, there are an abundance of available guidelines [11–19]. Health professional organizations like the American Diabetes Association (ADA) and the National Institute for Health and Care Excellence (NICE) update their management guidelines regularly [20, 21]. In mainland China and Hong Kong, based on international guidelines on pregnancy and diabetes mellitus, contextual guidelines for GDM management have been established through expert consensus [22, 23]. As the most authoritative form, CPGs have the potential to influence the care delivered by a large number of healthcare providers and consequently the outcomes for patients, so it is universally acknowledged that the methodological quality of guidelines is very important and should be appraised [24, 25]. Our previous research found that, in general, the quality of GDM guidelines was relatively higher than that in the previous year [26], while the domains of Rigor of Development, Stakeholder Involvement and Editorial Independence of guidelines still needed to be improved.

However, methodological quality of guideline is not the only way to evaluate a guideline. Whether guidelines provide valid recommendations is an aspect of particular importance to practitioners. It is noted that there may be conflict between methodological quality and the validity of recommendations, and current guidelines differ substantially in their management recommendations [27]. Whether the recommendations are in accordance with evidence and whether the recommendations suit the local context are unknown. This makes it hard for the busy practitioners, confronted with conflicting guideline recommendations, to determine which guideline to follow [27]. Many researchers are aware of the fact that it is imperative to find a unified system for evaluating the validity of recommendations. However, little work has been done in this area. In order to better ascertain the best treatment for GDM women and whether recommendations in current guidelines are valid or not, extracting and appraising the content of current guidelines are crucial. Therefore, the aim of this study was to extract and evaluate the recommendations included in guidelines for GDM management using a recommendation matrix (details in another article under review).

## Methods

A search was conducted in CPGs for GDM management. The search strategy used the keywords “pregnancy”, “gravida*”, “conception”, “maternity”, “diabetes”, “hyperglycemia”, “insulin resistance”, “glucose intolerance”, “guideline”, “criteria”, “recommendation” and “standard”. Information sources were identified from the National Institute for Health and Care Excellence (NICE), New Zealand Guidelines Group (NZGG), Scottish Intercollegiate Guidelines Network (SIGN), China Medlive, American Diabetes Association (ADA), Canadian Diabetes Association (CDA), International Diabetes Federation (IDF), PubMed, Web of Science, Embase, China National Knowledge Infrastructure (CNKI), Wanfang Chinese Periodical Database and VIP Chinese Periodical Database. The eligibility criteria included: ①full guideline that were available in English or Chinese; ②guidelines which contained recommendations regarding GDM interventions; ③guidelines that were issued between 2009 and 2018. Two independent reviewers selected documents for inclusion and appraised the methodological quality with the Appraisal of Guidelines for Research & Evaluation (AGREE) II instrument.

Based on the quality evaluations, the reviewers summarized recommendations in guidelines and assessed the content of guidelines by establishing a “recommendation matrix” (Table 1 as an example). For each included document, we extracted the following information: title of guideline, author, development institute (e.g. government, special organization, etc.), year of publication, guideline type, methodological quality (appraised with AGREE II) and relevant recommendations. For all recommendations extracted, we assessed whether or not they explicitly recommended with the consistency across search strategies, selection of evidence, evidence interpretation and resulting recommendations. Each of the recommendations was rated on a seven-point scale (1-strongly inconsistent to 7-strongly consistent). A quality score was calculated in the same way used in AGREE II [28], that is, for each recommendation, the score was calculated by summing up all the scores of the individual items and by scaling the total as a percentage of the maximum possible score [28]. If the guideline provided more complete information, we also extracted supporting evidence and the evidence level if the evidence has been cited, and the likelihood of applying the recommendation in China. For all guidelines, the recommendations were divided into five domains, namely, diagnosis of GDM, prenatal care, intrapartum care, neonatal care and postpartum care.

**Table 1:** Recommendations Extraction (NICE guideline as an example)

Basic information		
Title of guideline		Diabetes in pregnancy: Management of diabetes and its complications from preconception to the postnatal period
Development institute		NICE
Publication year		Published 2008, updated 2015
Guideline type		Evidence-based guideline
Guideline methodology		Developed in accordance with the NICE guideline development process
Quality assessment of evidence and grading of strength of recommendations		GRADE system
Guideline Currency	Literature search date	2014.6
	Search strategy	A comprehensive literature search was performed
Methodological quality of guideline		
AGREE II scores	Domain 1. Scope and Purpose	100%
	Domain 2. Stakeholder Involvement	100%
	Domain 3. Rigor of Development	100%
	Domain 4. Clarity of Presentation	100%
	Domain 5. Applicability	100%
	Domain 6. Editorial Independence	100%
	Overall assessment	☑Recommend ☐Recommend with modifications ☐Would not recommend
Recommendation extraction and assessment		
Health questions		What are the target ranges for blood glucose in women with gestational diabetes during pregnancy?
Specific recommendation		Advice pregnant women with any form of diabetes to maintain their capillary plasma glucose below the following target levels, if these are achievable without causing problematic hypoglycaemia: 1) fasting: 5.3 mmol/L(#1) and 2) 1 h after meals: 7.8 mmol/L(#2) or 3) 2 h after meals: 6.4 mmol/L.(#3)
Strength of recommendation		☑Strong ☐Week
Supporting evidence		(#1) 1 secondary analysis of RCT data, 1 RCT, very low (#2) 1 retrospective cohort study, very low (#3) 1 secondary analysis of RCT data, very low
Consistency appraisal		Search strategy and selection of evidence ☐1 ☐2 ☐3 ☐4 ☐5 ☐6 ☑7
		Evidence and interpretation ☐1 ☐2 ☐3 ☐4 ☐5 ☐6 ☑7
		Interpretation and recommendation ☐1 ☐2 ☐3 ☐4 ☐5 ☐6 ☑7

Initially, two researchers (Yingfeng Zhou and Mengxing Zhang) independently analyzed one guideline with the recommendation matrix in order to identify the validation and feasibility of the tool before determine the final result. Then the final form was used to extract recommendations content from the other guidelines. Frequent communication occurred between two researchers throughout the process so as to maximize inter-rater reliability. Any disagreements were settled through consultation with the study groups.

Descriptive statistics were conducted in order to characterize the recommendation content. For quantitative data, the statistical analysis was performed using Microsoft Office 2013 and SPSS Version 25.0. The total number, percentages, and mean, and standard deviation were calculated to describe the consistency of recommendations. In addition, a radar chart was also used to identify features of recommendation consistency in different aspects.

This article is part of a guideline adaptation project. The Guideline Adaptation Project has been registered in the International Guideline Register Center (http://www.guidelines-registry.cn), Registration number: IPGRP-2016CN015.

## Results

### Characteristics of included guidelines

**Fig. 1:**
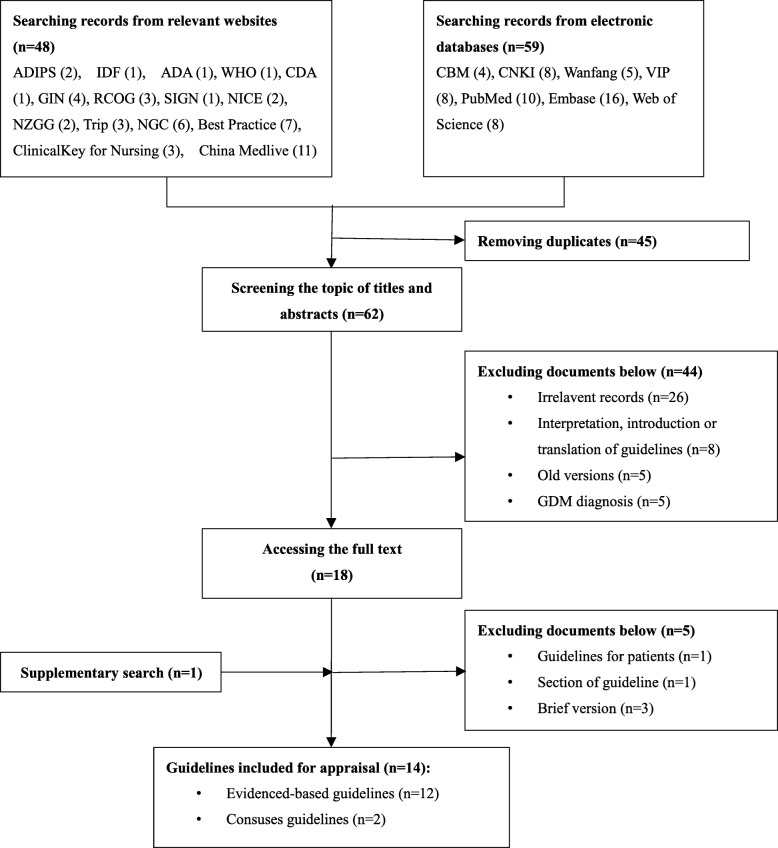
Flow chart of the systematic literature search and selection

Combining all searches yielded 108 relevant documents, of which 14 guidelines from international organizations were included: ADA (American Diabetes Association), NCC-WCH (National Collaborating Centre for Women’s and Children’s Health), IDF (International Diabetes Federation), FIGO (The International Federation of Gynecology and Obstetrics), CMA (Chinese Medical Association), DDG (German Diabetes Association), A.N.D. (Academy of Nutrition and Dietetics), API (The Association of Physicians of India), CDA (Canadian Diabetes Association), HKCOG (The Hong Kong College of Obstetricians and Gynecologists), American Endocrine Society, NZGG (New Zealand Guidelines Group), SIGN (Scottish Intercollegiate Guidelines Network), and Queensland Department of Health. See Fig. 1 for the flow diagram of the document selection process. Characteristics of the final included items are shown in Table 2.

**Table 2:** Characteristics of the 14 guidelines

Guidelines		Country/region	Development institute	Publication year	Type	Main content
1	Gestational Diabetes (2016) Evidence-Based Nutrition Practice Guideline [11]	USA	A.N.D.	2016	Evidence-based	The focus of this guideline is on nutrition practice during the treatment of women with GDM. Topics include: ①Referral to an RDN; ②Nutrition Assessment; ③MNT; ④Calories; ⑤Macronutrients; ⑥Vitamins and Minerals; ⑦Meal and Snack Distribution; ⑧High-Intensity Sweeteners; ⑨Alcohol; ⑩Physical Activity; ⑪Nutrition Monitoring and Evaluation
2	Clinical Practice Guidelines: Diabetes and Pregnancy [12]	Canada	CDA	2013	Evidence-based	“Diabetes and Pregnancy” is one of chapters of the full guideline--“Clinical Practice Guidelines”, which contains Pregestational Diabetes and GDM. GDM topics include: ①Screening and diagnosis; ②Management (Lifestyle, Glycemic control, Monitoring, Pharmacological therapy, Intrapartum glucose management, Intrapartum insulin management, Postpartum care, Planning future pregnancies)
3	Diabetes and Pregnancy: An Endocrine Society Clinical Practice Guideline [13]	USA	Endocrine Society	2013	Evidence-based	The Guideline addresses important clinical issues in the contemporary management of women with Pregestational Diabetes and women with GDM during and after pregnancy. GDM: ①Testing and diagnosis; ②Management of elevated blood glucose; ③Glucose monitoring and glycemic targets; ④Nutrition therapy and weight gain targets; ⑤Blood glucose-lowering pharmacological therapy during pregnancy, Labor, delivery, lactation, and postpartum care.
4	Global Guideline on Pregnancy and Diabetes [14]	International	IDF	2009	Evidence-based	The guideline is for pregnant women with known diabetes or GDM, and topics include: ①Pre-conception glycaemic control; ②Testing for GDM; ③Management during pregnancy (Monitoring glucose levels, Lifestyle management, Insulin use during pregnancy, Oral glucose-lowering agents in pregnancy); ④Management after pregnancy (Breastfeeding, Follow-up of GDM, Prevention of type 2 diabetes in women who developed GDM).
5	Screening, Diagnosis and Management of Gestational Diabetes in New Zealand: A clinical practice guideline [15]	New Zealand	NZGG	2014	Evidence-based	This guideline covers: ①Early screening of women for probable undiagnosed diabetes; ②Screening, diagnosis and management of women with GDM; ③Follow-up of women with GDM to detect type 2 diabetes after birth.
6	Queensland Clinical Guideline: Gestational diabetes mellitus [16]	Queensland	Department of Health	2015	Evidence-based	This guideline includes recommendations about: ①Risk Assessment of GDM; ②Antenatal Care (Maternal and Fetal surveillance, Psychosocial support, Self-monitoring, Medical nutrition therapy, Physical activity); ③Pharmacological therapy; ④Birthing Care; ⑤Postpartum care.
7	Management of diabetes: A national clinical guideline [17]	Scotland	SIGN	2013	Evidence-based	This guideline provides recommendations based on current evidence for best practice in the management of diabetes. “Management of diabetes in pregnancy” is one of updated chapters, which only contains a few recommendations about pre-pregnancy care, nutritional management, optimization of glycemic control, complication during pregnancy, fetal assessment, gestational diabetes, delivery, postnatal care.
8	Initiative on gestational diabetes mellitus: A pragmatic guide for diagnosis, management, and care [18]	International	FIGO	2015	Evidence-based	To address the issue of GDM, FIGO recommends the following: ①Public health focus; ②Universal testing; ③Criteria for diagnosis; ④Diagnosis of GDM; ⑤Management of GDM; ⑥Lifestyle management; ⑦Pharmacological management; ⑧Postpartum follow-up and linkage to care.
9	Consensus Evidence-based Guidelines for Management of Gestational Diabetes Mellitus in India [19]	India	API	2014	Evidence-based	The guideline presents an overview of following consensus: ①Screening for GDM; ②Diagnostic criteria for GDM; ③Blood glucose targets and monitoring; ④Oral anti-diabetic drugs; ⑤Insulin therapy; ⑥Continuous subcutaneous insulin infusion.
10	Standards of medical care in diabetes −2018 [20]	USA	ADA	2018	Evidence-based	It is a general Standards of Medical Care in Diabetes. “Management of Diabetes in Pregnancy” is a chapter of this guideline, which include following relevant recommendations: ①Preconception counseling; ②Glycemic targets in pregnancy; ③Management of GDM; ④Pregnancy and drug consideration
11	Diabetes in pregnancy: management from preconception to the postnatal period [21]	England	NICE, NCC-WCH	2015	Evidence-based	The guideline focus on Management of diabetes and its complications from preconception to the postnatal period: ①Preconception planning and care; ②Gestational diabetes; ③Antenatal care for women with diabetes; ④Intrapartum care; ⑤Postnatal care.
12	Gestational Diabetes Mellitus (GDM) – Diagnosis, Treatment and Follow-Up. Guideline of the DDG and DGGG [29]	Germany	DDG, DGGG	2018	Evidence-based	This guideline focus on: ①Screening and diagnosis; ②Treatment (First medical consultation after GDM diagnosis; Physical activity; Dietary counselling; Recommended weight gain; Blood glucose monitoring; Insulin therapy; Oral antidiabetic drugs and GLP-1 analogues); ③Obstetric care; ④Postpartum care.
13	Guidelines for the Management of Gestational Diabetes Mellitus [22]	Hong Kong	HKCOG	2016	Expert Consensus	This is an Expert Consensus focus on:①Diagnostic criteria and classification; ②Screening for hyperglycemia in pregnancy; ③Early detection of GDM and screening for pre-GDM in the first trimester; ④Management for hyperglycemia first detected in pregnancy; ⑤Postnatal management.
14	Diagnosis and Management of diabetes in pregnancy: A clinical practice guideline (2014) [23]	China	CMA	2014	Expert Consensus	This is an Expert Consensus focus on:①Diagnosis of GDM and PGDM; ②surveillance during pregnancy; ③counseling and treatment; ④Timing and mode of delivery; ⑤Postnatal management.

According to systematically evaluation with AGREE II instrument, the methodological quality of guidelines included varied. But generally, they scored well. Scores for six AGREE II domains (Mean ± SD) were:88% ± 0.15 (Scope and Purpose), 73% ± 0.30 (Stakeholder Involvement), 60% ± 0.29 (Rigor of Development), 89% ± 0.19 (Clarity of Presentation), 70% ± 0.34 (Applicability), 70% ± 0.41 (Editorial Independence).

### Comparison and summary of recommendations

Using the recommendation matrix, all relevant guideline information and recommendations included were extracted, and all health questions of each guideline were placed in the recommendation matrixes (Additional files 1, 2, 3, 4, 5, 6, 7, 8, 9, 10, 11, 12, 13 and 14). For example, we extracted the NICE guideline, which is displayed in Table 1. The NICE guideline was developed based on evidence, and the development process was distinctly clarified. The guideline group graded evidence and recommendations by Grading of Recommendations Assessment, Development and Evaluation (GRADE) system. With regard to health question “target blood glucose values”, the results of recommendation appraisal revealed high consistency in search strategy and selection of evidence, evidence and interpretation, as well as interpretation and resulting recommendations.

The effectiveness categorization of each domain based ons the recommendations was presented in Table 3. The similarities and differences between the different guidelines on each domain were discussed below.

**Table 3:** Recommendations summary

Health questions	Description	Guideline	Recommendations (example)
Diagnosis of GDM			
Risk factors	Factors that make pregnant women more likely to get GDM and should be recognized	2 evidence-based guidelines (NICE, CDA) 2 expert consensus (HKCOG, CMA)	Assess risk of gestational diabetes using risk factors in a healthy population. At the booking appointment, determine the following risk factors for gestational diabetes: ①BMI above 30 kg/m^2^; ② previous macrosomia baby weighing 4.5 kg or above; ③ previous gestational diabetes; ④ family history of diabetes (first-degree relative with diabetes); ⑤ minority ethnic family origin with a high prevalence of diabetes.
Screening	Screening method to identify women who have GDM	9 evidence-based guidelines (NICE, NZGG, SIGN, ADA, FIGO, NGC, CA, API, IDF) 2 expert consensus (HKCOG, CMA)	Use the 2-h 75 g oral glucose tolerance test (OGTT) to test for gestational diabetes in women with risk factors. Offer women with any of the other risk factors for gestational diabetes a 75 g 2-h OGTT at 24–28 weeks.
Diagnostic criteria	Diagnostic criteria for GDM	7 evidence-based guidelines (SIGN, ADA, FIGO, NGC, A.N.D., DDG Queensland) 2 expert consensus (HKCOG, CMA)	GDM should be diagnosed at any time in pregnancy if one or more of the following criteria are met following a 75 g glucose load: ① fasting PG 5.1–6.9 mmol/l; ② 1-h PG ≥ 10.0 mmol/l; ③ 2-h PG 8.5–11.0 mmol/l
Prenatal Care			
Health education	Inform women with GDM relevant information	7 evidence-based guidelines (NICE, NZGG, SIGN, ADA, FIGO, IDF, A.N.D.) 1 expert consensus (CMA)	Explain that:① in some women, gestational diabetes will respond to changes in diet and exercise; ② the majority of women will need oral blood glucose-lowering agents or insulin therapy if changes in diet and exercise do not control gestational diabetes effectively; ③ if gestational diabetes is not detected and controlled, there is a small increased risk of serious adverse birth complications such as shoulder dystocia; ④ a diagnosis of gestational diabetes will lead to increased monitoring, and may lead to increased interventions, during both pregnancy and labor.
Medical nutrition therapy	Medical nutrition therapy (MNT) recommendations for management of GDM that assist in achieving and maintaining glycemia, and reducing the risk of adverse maternal and neonatal outcomes	11 evidence-based guidelines (NICE, NZGG, SIGN, ADA, FIGO, NGC, CDA, API, IDF, Queensland, A.N.D.) 2 expert consensus (HKCOG, CMA)	In women with GDM, the registered dietitian nutritionist (RDN) should provide adequate amounts of macronutrients to support pregnancy, based on nutrition assessment, with guidance from the Dietary Reference Intakes (DRI).
Physical activity	Physical activity recommendations for management of GDM.	6 evidence-based guidelines (NICE, ADA, FIGO, NGC, IDF, DDG) 2 expert consensus (HKCOG, CMA)	Advice regular exercise (such as walking for 30 min after a meal) to improve glycemic control.
Pharmacological therapy	Pharmacological therapy for management of GDM, including insulin and oral hypoglycemic agents	5 evidence-based guidelines (ADA, CDA, API, IDF, DDG) 1 expert consensus (CMA)	For women who are non-adherent to or who refuse insulin, glyburide or metformin may be used as alternative agents for glycemic control.
Blood glucose monitoring	Effect blood glucose monitoring method in predicting adverse outcomes in women with GDM	9 evidence-based guidelines (NICE, SIGN, ADA, FIGO, NGC, CDA, API, IDF, Queensland,) 2 expert consensus (HKCOG, CMA)	Self-monitoring of blood glucose is recommended for all pregnant women with diabetes, 3–4 times a day: • Fasting: once daily, following at least 8 h of overnight fasting • Postprandial: 2–3 times daily, 1 or 2 h after the onset of meals, rotating meals on different days of the week
Target blood glucose values	Target ranges for blood glucose in women with GDM	7 evidence-based guidelines (NICE, NZGG, ADA, FIGO, NGC, CDA, API) 2 expert consensus (HKCOG, CMA)	Targets for glucose control during pregnancy: • Fasting glucose < 5.3 mmol/L • 1-h postprandial < 7.8 mmol/L • 2-h postprandial < 6.7 mmol/L
Ketone monitoring	Ketone monitoring and target ranges in pregnancy in women with GDM	1 evidence-based guidelines (NICE) 1 expert consensus (CMA)	Test urgently for ketoaemia if a pregnant woman with any form of diabetes presents with hyperglyaemia or is unwell, to exclude diabetic ketoacidosis.
HbA1c monitoring	HbA1c monitoring and target ranges in pregnancy in women with GDM	2 evidence-based guidelines (NICE, IDF) 1 expert consensus (CMA)	Use HbA1c as an ancillary aid to self-monitoring. Aim for an HbA1c < 6.0%, or lower if safe and acceptable.
Continuous glucose monitoring	continuous glucose monitoring recommendations during pregnancy	3 evidence-based guidelines (NICE, NGC, API) 1 expert consensus (CMA)	Do not offer continuous glucose monitoring routinely to pregnant women with diabetes.
Fetal monitoring	Screening for congenital malformations and monitoring fetal growth and wellbeing	4 evidence-based guidelines (NICE, NZGG, SIGN, FIGO) 1 expert consensus (CMA)	Offer women with GDM an ultrasound scan at the time of diagnosis and at 36–37 weeks. Further ultrasound scans should be based on clinical indications. Treatment decisions should not be based solely on fetal ultrasound.
Intrapartum Care			
Timing and mode of birth	Optimal timing and mode of birth in women with GDM	4 evidence-based guidelines (NICE, NZGG, SIGN, FIGO) 1 expert consensus (CMA)	Discuss the timing and mode of birth with pregnant women with diabetes during antenatal appointments, especially during the third trimester.
Glycemic control	Maintaining maternal blood glucose in target range during labor and birth to reduce the incidence of neonatal hypoglycemia and reduce fetal distress.	6 evidence-based guidelines (NICE, SIGN, FIGO, NGC, CDA, API) 1 expert consensus (CMA)	Women should be closely monitored during labor and delivery, and maternal blood glucose levels should be kept between 4.0 and 7.0 mmol/L in order to minimize the risk of neonatal hypoglycemia.
Neonatal Care			
Neonatal hypoglycemia	Prevention, assessment and treatment of neonatal hypoglycemia	3 evidence-based guidelines (NICE, NZGG, SIGN) 1 expert consensus (CMA)	Measure the infant’s plasma glucose at 1–2 h of age, 4 h, and then 4-hourly, preferably before feeds, until there have been three consecutive readings > 2.6 mmol/L.
Initial assessment	Neonatal assessment and criteria for admission to intensive or special care	2 evidence-based guidelines (NICE, NGC) 1 expert consensus (CMA)	Carry out blood glucose testing routinely in babies of women with diabetes at 2–4 h after birth. Carry out blood tests for polycythemia, hyperbilirubinemia, hypocalcemia and hypomagnesemia for babies with clinical signs.
Postpartum Care			
Blood glucose control	Including taking insulin, oral hypoglycemic agents to control blood glucose and using other medicines, as well as breastfeeding after birth	6 evidence-based guidelines (NICE, NZGG, NGC, CDA, API, IDF) 2 expert consensus (HKCOG, CMA)	Women should be encouraged on breastfeeding. They can resume or continue to take metformin and glibenclamide immediately after birth as required, but should avoid other forms of oral hypoglycemic agents while breastfeeding.
Information and follow-up	Education interventions after delivery	8 evidence-based guidelines (NICE, NZGG, SIGN, ADA, FIGO, NGC, IDF, Queensland) 2 expert consensus (HKCOG, CMA)	Women diagnosed with hyperglycemia in pregnancy should be informed about the increased risk of future DM and hyperglycemia in future pregnancy and should be offered lifestyle advice including weight control, diet and exercise.
Postnatal blood glucose testing	Accuracy and timing of postnatal blood glucose testing in women who had GDM	8 evidence-based guidelines (NICE, NZGG, SIGN, ADA, NGC, CDA, IDF, DDG) 2 expert consensus (HKCOG, CMA)	Offer a postnatal test at 6–12 weeks to exclude DM, either OGTT or HbA1c (with or without fasting glucose).

#### Diagnosis of GDM

The first domain was diagnosis of GDM, which covered three health questions: risk factors of GDM, GDM screening and diagnostic criteria. Risk factors for GDM were identified in five guidelines [12, 21–23, 29], mainly including personal and family history, relevant medical history, past pregnancy and current history. It was noted that threshold of some risk factors were discrepant in different guidelines. As an example, advanced maternal age, obesity BMI and macrosomia weighing in Hong Kong College of Obstetricians and Gynaecologists (HKCOG) guideline [22] had a much smaller value then in western countries. NICE guideline recommended that pregnant women with risk factors should be screened, while other guidelines recommended that universal screening was preferred. As to diagnostic criteria, the International Association of the Diabetes and Pregnancy Study Groups (IADPSG) (2010) criteria was adopted by most guidelines. In this study, eight guidelines [11, 13, 16–18, 22, 23, 29] included used IADPSG (2010) criteria, recommending that GDM should be diagnosed at any time in pregnancy if one or more of the following criteria were met following a 75 g oral glucose tolerance test (OGTT): 1) fasting PG 5.1–6.9 mmol/L; 2) 1-h PG ≥ 10.0 mmol/L; 3) 2-h PG 8.5–11.0 mmol/L, while six other guidelines recommended alternatives.

#### Prenatal care

Prenatal care was a very crucial domain of GDM management. All guidelines agreed that it was necessary to encourage GDM women to take prenatal care. All guidelines, excepting the A.N.D. guideline [11] that only mentioned nutrition therapy, made recommendations in similar aspects of prenatal interventions more or less, which might refer to health education, medical nutrition therapy, physical activity, pharmacological therapy, blood glucose monitoring, target blood glucose values, ketone monitoring, HbA1c monitoring, continuous glucose monitoring and fetal assessment. The main principles included: ①offer all women ongoing treatment by multidisciplinary health professionals once they were diagnosed; ②lifestyle intervention was a primary and essential component of management, especially nutrition therapy; ③medical therapy should be started if needed to achieve glycemic targets; and ④ self-monitoring of blood glucose regularly should be emphasized. However, recommendations of a similar theme were not always unanimous in different guidelines. For example, six guidelines [12, 14, 19, 20, 23, 29] recommended that insulin was the preferred medication for treating hyperglycemia in GDM. On the contrary, other six guidelines [13, 16–18, 21, 22] did not regard insulin as the first option when drug treatment was required, since it was proved that oral antidiabetic agents was safe and might even significantly reduce several adverse maternal and neonatal outcomes (Table 4). In addition, women’s preferences and the ability to adhere to medication and self-monitoring were also considered in different guidelines.

**Table 4:** Pharmacological therapy recommendations among different guidelines

Guidelines	Recommendation
NICE, 2015	① Offer metformin to women with gestational diabetes if blood glucose targets are not met using changes in diet and exercise within 1–2 weeks; ② Offer insulin instead of metformin to women with gestational diabetes if metformin is contraindicated or unacceptable to the woman; ③ Consider glibenclamide for women with gestational diabetes: in whom blood glucose targets are not achieved with metformin but who decline insulin therapy or who cannot tolerate metformin.
NZGG, 2014	Where women who have gestational diabetes and poor glycaemic control (above treatment targets) in spite of dietary and lifestyle interventions, offer oral hypoglycaemics (metformin or glibenclamide) and/or insulin therapy. In deciding whether to use oral therapy or insulin, take account of the clinical assessment and advice, and the woman’s preferences and her ability to adhere to medication and self-monitoring.
SIGN, 2013	Metformin or glibenclamide may be considered as initial pharmacological, glucose-lowering treatment in women with gestational diabetes.
ADA, 2018	Insulin is the preferred medication or treating hyperglycemia in gestational diabetes mellitus as it does not cross the placenta to a measurable extent. Metformin and glyburide may be used, but both cross the placenta to the fetus, with metformin likely crossing to a greater extent than glyburide. All oral agents lack long-term safety data.
FIGO, 2015	① Insulin, glyburide, and metformin are safe and effective therapies for GDM during the second and third trimesters, and may be initiated as first-line treatment after failing to achieve glucose control with lifestyle modification. Among OADs, metformin may be a better choice than glyburide; ② High resource: Insulin should be considered as the first-line treatment in women with GDM who are at high risk of failing on OAD therapy, including some of the following factors: • Diagnosis of diabetes < 20 weeks of gestation • Need for pharmacologic therapy > 30 weeks • Fasting plasma glucose levels > 110 mg/dL • 1-h postprandial glucose > 140 mg/dL • Pregnancy weight gain > 12 kg
Endocrine Society, 2013	① We suggest that glyburide (glibenclamide) is a suitable alternative to insulin therapy for glycemic control in women with gestational diabetes who fail to achieve sufficient glycemic control after a 1-week trial of medical nutrition therapy and exercise except for those women with a diagnosis of gestational diabetes before 25 weeks gestation and for those women with fasting plasma glucose levels > 110 mg/dl (6.1 mmol/l), in which case insulin therapy is preferred; ② We suggest that metformin therapy be used for glycemic control only for those women with gestational diabetes who do not have satisfactory glycemic control despite medical nutrition therapy and who refuse or cannot use insulin or glyburide and are not in the first trimester.
CDA, 2013	① If women with GDM do not achieve glycemic targets within 2 weeks from nutritional therapy alone, insulin therapy should be initiated; ② For women who are nonadherent to or who refuse insulin, glyburide or metformin may be used as alternative agents for glycemic control. Use of oral agents in pregnancy is off-label and should be discussed with the patient.
API, 2014	The use of OADs is currently not recommended for glycaemic management during pregnancy.
IDF, 2009	Insulin has been, and is likely to remain, the treatment of choice but there is now adequate evidence to consider the use of metformin and glibenclamide (glyburide) as treatment options for women who have been informed of the possible risks. Combination therapy has not been specifically studied.
Queensland, 2015	① Metformin when compared to Insulin is effective at lowering blood glucose and is safe for pregnant women and their fetuses; ②I nsulin is safe to use in pregnancy.
HKCOG, 2016	① Offer metformin if blood glucose targets are not met after diet and exercise therapy within 1–2 weeks; ② Offer addition of insulin to diet therapy, exercise and metformin if blood glucose targets are not met. ③ Consider glibenclamide for women in whom blood glucose targets are not achieved with metformin but who decline insulin therapy or who cannot tolerate metformin.
CMA, 2014	Insulin should be considered as the first-line treatment in women with GDM, and OADs is currently not recommended for glycaemic management during pregnancy.
DDG, 2018	① The indication for insulin should first be considered within 1–2 weeks after the start of basic therapy (diet, exercise); ② For pregnant women with GDM and suspected severe insulin resistance and when individually indicated, use of metformin can be considered following explanation of the off-label use.

#### Intrapartum care

The intrapartum care domain contained timing and mode of birth and glycemic control. Each guideline differed slightly on recommendations for timing and mode of birth, however, commonality in the way in which timing and mode of birth was decided was described, in other words, depending on whether there were maternal or fetal complications. Recommendations for glycemic control during labor and birth were similar for most guidelines, namely, monitoring capillary plasma glucose during labor and birth, and ensuring that it was maintained in normal glucose values (five guidelines [12, 13, 17, 18, 21] recommended to maintain blood glucose levels between 4 and 7 mmol/L).

#### Neonatal care

The fourth domain was neonatal care, that is, neonatal hypoglycemia and neonatal initial assessment. Only five guidelines [12, 15, 17, 21, 23] mentioned recommendations for neonatal hypoglycemia, advising to avoid neonatal hypoglycemia through measuring the infant’s plasma glucose frequently and early feeding. In addition, for newborns who had clinical signs associated with neonatal complications, NICE guidelines also made additional recommendations for neonatal initial assessment and criteria for admission to intensive or special care.

#### Postpartum care

Postpartum care was a domain involving medicines and breastfeeding after delivery, information and follow-up after birth and postnatal testing. Most guidelines recommended that GDM women should discontinue blood glucose-lowering therapy immediately after birth, but HKCOG guidelines [22] emphasized that those women could also resume or continue to take metformin and glibenclamide after birth as required. Early and exclusively breastfeeding was highly encouraged, for its benefits for both mother and infant. Regarding postnatal education, it was unanimously agreed in all guidelines that women diagnosed with GDM should be informed of the increased risk of GDM in a subsequent pregnancy and the increased risk for developing type 2 diabetes. Hence, it was important to provide them with advice on how to maintain a healthy lifestyle and information on postnatal testing. Recommendations for postnatal testing were slightly different. The method of postnatal testing can be OGTT or HbA1c (with or without fasting glucose). And testing time ranged from the initial month to 6 months, mainly between six to 12 weeks after birth. Then assessment of glycemia using fasting glucose or HbA1c should be carried out at regular intervals thereafter.

### Assessment of consistency

**Fig. 2:**
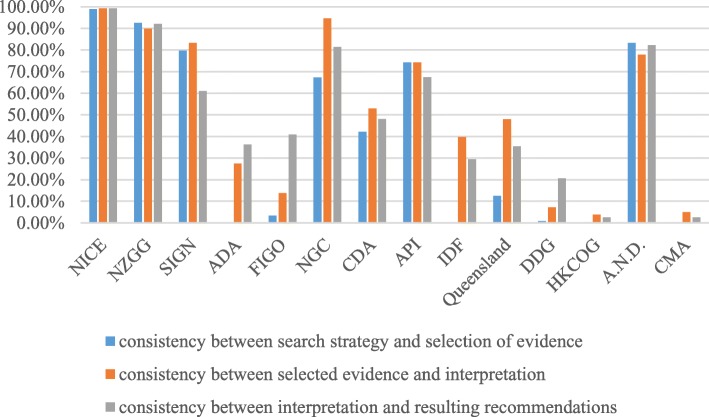
Consistency appraisal of guidelines

**Fig. 3:**
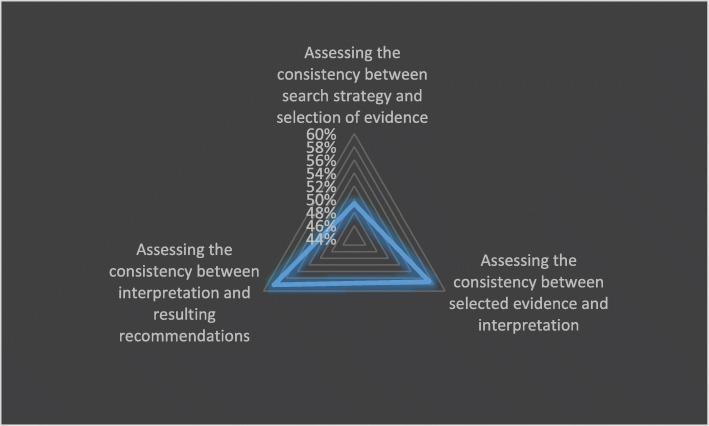
Consistency appraisal in all recommendations

A total of 361 original recommendations for GDM management which were from 14 guidelines were included. Although some recommendations did not fall into any of the identified themes, we undertook consistency appraisal of these as well. As presented in Table 5, different guidelines appeared to have significant discrepancies in consistency of guideline content. Even in the same guideline, consistency differed in three aspects: ①consistency between search strategy and selection of evidence, ②consistency between selected evidence and interpretation, and ③consistency between interpretations and resulting recommendations. Among all guidelines included, NICE guidelines showed the best average score of consistency in each aspect. However, HKCOG guidelines and CMA guidelines received extremely low scores in each aspect. Apparently, in this study, evidence-based guidelines rated relatively higher in content consistency than expert consensus-based guidelines. Consistency appraisal of each guideline is presented in Fig. 2. For consistency in each aspect, most guidelines showed the same tendency, that is, a guideline which received high average scores could also receive high scores in the other two aspects, and, conversely, low average scores in all aspects. When it came to all recommendations, search strategy and selection of evidence were slightly inconsistent. The radar chart showing comparable consistency between search strategy and selection of evidence, between selected evidence and interpretation, and between interpretation and resulting recommendations (scilicet 49.31, 57.20 and 58.17%, respectively) is presented in Fig. 3.

**Table 5:** Consistency characteristics of guidelines

Guidelines	N	Mean (SD)		
		C1*	C2*	C3*
NICE	74	6.93 (0.34)	6.96 (0.26)	6.96 (0.26)
NZGG	38	6.55 (0.76)	6.39 (0.82)	6.53 (0.65)
SIGN	18	5.78 (1.11)	6.00 (0.91)	4.67 (0.59)
ADA	17	1.00 (0.00)	2.65 (1.17)	3.18 (1.24)
FIGO	40	1.20 (0.72)	1.83 (1.65)	3.45 (2.33)
Endocrine Society	25	5.04 (1.72)	6.68 (1.25)	5.88 (1.81)
CDA	17	3.53 (2.43)	4.18 (2.40)	3.88 (1.69)
API	22	5.45 (2.22)	5.45 (2.22)	5.04 (2.38)
IDF	13	1.00 (0.00)	3.38 (2.29)	2.77 (1.24)
Queensland	8	1.75 (0.71)	3.88 (1.36)	3.13 (1.25)
HKCOG	13	1.00 (0.00)	1.23 (0.60)	1.15 (0.55)
A.N.D.	15	6.00 (0.00)	5.67 (0.49)	5.93 (0.26)
DDG	21	1.05 (0.22)	1.43 (0.75)	2.24 (1.30)
CMA	40	1.00 (0.00)	1.30 (0.72)	1.15 (0.48)
Total	361	4.00 (2.74)	4.43 (2.59)	4.49 (2.42)

C1*: consistency between search strategy and selection of evidence

C2*: consistency between selected evidence and interpretation

C3*: consistency between interpretation and resulting recommendations

## Discussion

Gestational diabetes mellitus is a challenging complication of pregnancy that many women and doctors struggle with. In this review, we examined the existing guidelines on the management for GDM in 11 countries or regions. Given that appropriate methodologies and rigorous strategies in the guideline development process are crucial for guideline implementation [25], the development methods of the guidelines were measured using the AGREE II instrument. In general, the quality of GDM guidelines, especially evidence-based guidelines, was high. This could be explained by the fact that much progress has been made in the development of methodological and reporting criteria of evidence-based guidelines within the past decade [30]. Nonetheless, as the results in previous study revealed, the domains of Rigor of Development, Stakeholder Involvement and, Editorial Independence still need to be improved [26].

It is noted that practice guidelines with the best methodological quality were not necessarily the most valid in their recommendations [27]. Thus it is important to emphasize that clinical practitioners should critically evaluate the methodological quality as well as the content of the recommendations before adopting the recommendations, which leads to another issue, that is, consistency appraisal. Despite many researchers being aware of the crucial role of the appraisal of consistency between evidence and resulting recommendations, there are no existing criteria for assessing content consistency of guidelines. In guideline adaptation of some topics, qualitative analysis was used in content extraction, which formulated a general description of the research topic through generating categories without any consistency appraisal [31, 32]. In this review, we developed a “recommendation matrix” on the basis of the CAN-Implement© method [33], and used the tool to extract and assess guideline content. As a recommendation matrix was used, not only relevant and potentially relevant recommendations on all pre-specified healthcare aspects for GDM care were identified, but also consistency between search strategy and selection of evidence, between selected evidence and interpretation, and between interpretation and resulting recommendations was assessed. The results showed that current guidelines on GDM care are of varied consistency, and guidelines developed in internationally recognized guideline development methodology show better consistency. Also guidelines that have low consistency in one aspect may also have low consistency in other two aspects. This is probably because reporting quality of guidelines is the cornerstone of consistency assessment. Those guidelines with evidence tables or technical reports not published may also show low consistency. Thus, guideline development committees are strongly encouraged to make use of guideline development manuals when drafting guidelines.

Regarding guideline content, five aspects were analyzed: diagnosis of GDM, prenatal care, intrapartum care, neonatal care, and postpartum care. Most recommendations in guidelines focused on prenatal care, especially all kinds of therapies that might reduce the risk of adverse pregnancy outcomes related to uncontrolled blood sugar pre-conception. This review generated similar results with those from a previous study that international guidelines were consistent in most of their recommendations [34]. Nonetheless, although commonality in most areas existed, there were still some discrepancies among guidelines. For example, recommendations regarding oral hypoglycemic agents in the guidelines diverged. Some guidelines recommended that oral hypoglycemic agents be considered as an initial pharmacological intervention, while some guidelines only considered insulin as an exclusive hypoglycemic medicine. Guidelines were supported with evidence, so inconsistency may be caused by insufficient evidence on pharmacological interventions in the period in which the guidelines were developed [26]. However, it should be reminded that even though all evidence available was identified, consensus usually did not warrant similar recommendations in different contexts. This was because when a recommendation was developed, not only available evidence, but benefits and harms, patients’ values and preferences, as well as resource implications, should be appropriate considered [10].

Since recommendations were well summarized, guideline adaptation was required to maintain the validity of recommendations in different health care systems. Guideline adaptation involves using knowledge synthesis of existing guidelines to produce recommendations, rather than relying only on a review of primary literature, for the purpose of reducing duplication of effort [35]. In mainland China and Hong Kong, there were only expert consensus for GDM care [22, 23], without a national GDM management evidence-based guideline adapted to the Chinese context previously. In this instance, it is recommended to adapt the clinical practice guideline related to GDM management for the local context, providing support for professionals to make better decisions in clinical practice. How to select, tailor and implement recommendations and supporting evidence extracted is the next challenging step.

## Limitation

Due to the language barriers, we only included guidelines in English and Chinese. As a result, we only got existing guidelines on the management for GDM in 11 countries or regions in this review. And yet, we have no idea whether other countries use the recommendations provided by a certain guideline or use recommendations developed in their own language.

Another key limitation of this study is the subjectivity in appraising the consistency between evidence and recommendations. Although we attempted to minimize these discrepancies by stating the assessment criteria and through rigorous discussion, the results of the consistency appraisal still varied because of different understandings between researchers. Additionally, reporting quality of some guidelines is not clear cut, which was another barrier in the process of content analysis. Apart from this, this is the first time that we used a “recommendation matrix” in content analysis, and the tool we developed may still need to be modified.

## Conclusion

This paper describes the process used to extract and access the content of guidelines for GDM management. In conclusion, the recommendations were developed in five aspects: diagnosis of GDM, prenatal care, intrapartum care, neonatal care and postpartum care. The consistency of guidelines on the management of GDM in pregnancy is highly variable and this inconsistency needs to be addressed. Also, this review has proven that a “recommendation matrix” can be a tool to extract and assess consistency of guidelines. Additionally, our findings indicated that it is necessary to adapt and disseminate easily understandable evidence-based guidelines based on knowledge synthesis of existing guidelines in this paper.

## Additional files

Additional file 1: Recommendations Extraction of NICE guideline. The NICE guideline was developed in accordance with the NICE guideline development process. There were 74 relevant recommendations being extracted, which were displayed and appraised in Additional file 1. (XLSX 24 kb)

Additional file 2: Recommendations Extraction of NZGG guideline. The NZGG guideline development team followed a structured process for guideline development, and there were 38 relevant recommendations being extracted, which were displayed and appraised in Additional file 2. (XLSX 19 kb)

Additional file 3: Recommendations Extraction of SIGN guideline. The SIGN guideline was developed using a standard methodology according to SIGN guideline manual. There were 18 relevant recommendations being extracted, which were displayed and appraised in Additional file 3. (XLSX 16 kb)

Additional file 4: Recommendations Extraction of ADA guideline. The ADA guideline was developed using a standard methodology by the ADA’s Professional Practice Committee. The guideline included general recommendations about all kinds of diabetes, and there were only 17 relevant recommendations being extracted, which were displayed and appraised in Additional file 4. (XLSX 15 kb)

Additional file 5: Recommendations Extraction of FIGO guideline. The FIGO brought together international experts to develop the guideline, and suggestions are provided for a variety of different regional and resource settings. There were 40 relevant recommendations being extracted, which were displayed and appraised in Additional file 5. (XLSX 19 kb)

Additional File 6: Recommendations Extraction of Endocrine Society guideline. The Endocrine Society guideline was searched on the NGC website, which provided recommendations for the management of the pregnant woman with diabetes. Twenty-five relevant recommendations were extracted and appraised, which were displayed in Additional file 6. (XLSX 16 kb)

Additional file 7: Recommendations Extraction of CDA guideline. The CDA guideline was developed following the process used to develop previous Canadian Diabetes Association clinical practice guidelines, and AGREE II were incorporated into the guideline development process. There were 17 relevant recommendations being extracted, which were displayed and appraised in Additional file 7. (XLSX 15 kb)

Additional file 8: Recommendations Extraction of API guideline. To develop API guideline, existing guidelines, meta-analyses, cross sectional studies, systematic reviews and key cited articles were reviewed, and the recommendations were discussed at the national insulin summit. There were 22 relevant recommendations being extracted, which were displayed and appraised in Additional file 8. (XLSX 17 kb)

Additional file 9: Recommendations Extraction of IDF guideline. The guideline was developed through a non-formal evidence review and discussed by a small Writing Group. There were 13 relevant recommendations being extracted, which were displayed and appraised in Additional file 9. (XLSX 14 kb)

Additional file 10: Recommendations Extraction of Queensland guideline. The Queensland guideline was developed based on evidence, and there were 8 relevant recommendations being extracted, which were displayed and appraised in Additional file 10. (XLSX 14 kb)

Additional file 11: Recommendations Extraction of HKCOG guideline. The HKCOG guideline was an expert consensus. It was updated taking reference to the recent evidence, WHO, NICE guideline and recommendations of other international bodies. There were 13 relevant recommendations being extracted, which were displayed and appraised in Additional file 11. (XLSX 14 kb)

Additional file 12: Recommendations Extraction of A.N.D. guideline. The guideline focused on nutrition practiece during the treatment of women with GDM. There were 15 relevant recommendations being extracted, which were displayed and appraised in Additional file 12. (XLSX 15 kb)

Additional file 13: Recommendations Extraction of DDG guideline. The recommendations of DDG guideline were based on the evidence from the literature, which was selected through a systematic external literature search. There were 21 relevant recommendations being extracted, which were displayed and appraised in Additional file 13. (XLSX 15 kb)

Additional file 14: Recommendations Extraction of CMA guideline. The CMA guideline was an expert consensus. There were 40 relevant recommendations being extracted, which were displayed and appraised in Additional file 14. (XLSX 19 kb)

## Abbreviations

A.N.D: Academy of Nutrition and Dietetics
ADA: American Diabetes Association
API: The Association of Physicians of India
CDA: Canadian Diabetes Association
CMA: Chinese Medical Association
DDG: German Diabetes Association
DGGG: German Gynecology and Obstetrics Association
FIGO: The International Federation of Gynecology and Obstetrics
HKCOG: The Hong Kong College of Obstetricians and Gynaecologists
IDF: International Diabetes Federation
NCC-WCH: National Collaborating Centre for Women's and Children's Health
NGC: National Guideline Clearinghouse
NICE: The National Institute for Health and Care Excellence
NZGG: New Zealand Guidelines Group
SIGN: Scottish Intercollegiate Guidelines Network
WHO: World Health Organization
